# The Effect of Nano Calcium Carbonate and/or Recombinant Bone Morphogenetic Protein as a Biological Orthodontic Retainer on the Body Weight of Experimental Rat

**DOI:** 10.7759/cureus.34200

**Published:** 2023-01-25

**Authors:** Ata’a G Abdalameer, Hayder F Saloom

**Affiliations:** 1 Orthodontics, College of Dentistry, Mustansiriya University, Baghdad, IRQ; 2 Orthodontics, College of Dentistry, University of Baghdad, Baghdad, IRQ

**Keywords:** nanoparticles, recombinant, body weight, calcium carbonate, biological orthodontic

## Abstract

Background: Retention after orthodontic tooth movement (OTM) is essential to prevent relapse. This study examined the effects of a fixed orthodontic device and Nano Calcium Carbonate (CaCO_3_) nanoparticles with or without recombinant human bone morphogenetic protein (rhBMP) on rat body weight.

Materials and Methods: OTM was administered for 21 days to 80 Wistar Albino rats. First molar mesialization was active then forming two 40-rat groups which were subdivided into four subgroups of 10 rats each. These subgroups received 5 µg/kg rhBMP, 75 µg/kg CaCO_3_, 80 µg/kg rhBMP-loaded CaCO_3_ and one control. The relapse rate was examined weekly over the second 21 days when the second group exhibited mechanical retention and the first did not. Group 1 rats were murdered after 21 days (day 42), whereas group 2 rats entered a third 21-day post-retention period and then murdered (day 63). BW and OTM were measured on days 1, 21, 28, 35, 42, and 63.

Results: Within each group, the animal body weight was reduced significantly after the intervention and continued over time with a higher average reduction in the 9-week group than the 6-week group. However, there were no significant (P-value ˃0.05) differences in the BW between the groups of the two (6-week and 9-week) sets and the subgroups of the 6-week set across each time point. In contrast, there was a significant (P-value ˂0.05) difference between the BW of the conjugate subgroup and the other three subgroups in the 9-week set, particularly on 63^rd^ day.

Conclusion: CaCO_3_ nanoparticles and/or BMP with orthodontic treatment collectively or individually cause a reduction of body weight in rats.

## Introduction

After completing orthodontic tooth movement (OTM), relapse is a significant problem that can be happened in future. After orthodontic treatment, the only way to preserve teeth in their new locations and avoid relapse is with mechanical retention, which holds teeth in place while supporting tissues recover. Biotechnology can speed up the healing process of tissues and eliminate the need for mechanical retention [1]. Over the past 20 years, there has been a rise in the use of biological orthodontics with a number of biological treatments. The processes including osteoprotegerin, relaxion, and bone morphogenic proteins (BMPs), have been investigated for their potential to stop tooth movement and increase postorthodontic stability. BMPs that produce bone, cartilage, and platelet-derived matrix [2]. In orthodontics, the use of BMPs led to enhanced incisor stability and tissue regeneration [3]. The material is osteoconductive, biocompatible, and biodegradable and also inexpensive. This material is suitable for use in controlled medication release applications due to the fact that its disintegration is slow and that it is pH sensitive [4,5]. Researchers believe CaCO_3_ nanoparticles might be used in medical treatment and the clinical or histological consequences of biological retainers used in orthodontic treatment should be investigated. Rats were used to study the anatomy, physiology, pathology, and pharmacology of both animals and humans [6,7]. The practice of orthodontics can improve a ’patient’s self-esteem, facial appearance, oral health, and work performance; however, the user may experience mild to severe pain [8,9]. Patients should stay away from meals that are dense, sticky, and fibrous in order to prevent damage to their orthodontic devices, dental caries, and poor oral hygiene. Concerns about dietary limitations and possible weight loss during orthodontic treatment are common among patients and these factors may impair patient compliance [10,11]. According to the findings of several studies, orthodontic treatment may restrict the food options available to patients as a means of relieving their pain and suffering. At the clinical level, previous literature showed that weight loss in experimental animals is multifactorial [12]. However, no previous study investigated and compared the effect of local injection of biological retainers (Nano CaCO_3_ and/or rhBMPs) post active and passive orthodontic appliance on the body weight (BW) of rats. Thus, the study was carried out to assess the effect of CaCO_3_ NPs and/or rhBMPs on the BW of rats after they were treated with active and/or passive orthodontic devices.

## Materials and methods

Ethics and sampling

Ethical approval for this study was granted by the Animal Research Ethics Committee of the College of Dentistry/ University of Baghdad (Ref. Number: 179, Date 16/1/2020). The sample size was calculated to use 80 rats for this study based on a study conducted to measure tooth movement distance and relapse after active OTM [13]. They considered a sample size of 10 rats per group to be sufficient to detect a statistically significant difference with good power estimation.

Animals housing and feeding

This study was conducted at the National Center for Drug Control and Research/Ministry of Health/Baghdad-Iraq. The 80 rats (Albino Wistar) of male gender weighing 350 to 500 g were indicated in this research. All animals were housed for three weeks prior to the experiment commencement in cages (one rat/cage) in a room with 12 hours of dark and light cycles. The animals were provided with rat pellets and ad libitum. Rats were supplied with a softened rodent diet (granular diet dissolved in water) one week before the experiment till the day of sacrificing.

Orthodontic appliance in situ

Firstly, each rat BW was measured using a digital balance (max. capacity 1000 g, Kent scientific Cooperation, USA) to determine the dose of general anesthetic agents (intramuscular injection of a combination of ketamine, 8 mg/kg and xylazine, 5 mg/kg). All rats were inspected for a complete and intact set of teeth before any treatment. OTM was achieved using the active appliance designed by Kaipatur with some modifications [14].

In brief, it composed of a 9 mm NiTi closed coiled spring (GAC International, Bohemia, NY, USA) secured to a left first molar ligated wire. The other end of the spring was tightly secured to the head of a temporary skeletal anchorage device (micro-implant, 𝜇implant 1.2 × 3 mm in diameter self-threading Stryker-Leibinger Inc., Hamilton, ON, Canada). Accurate 𝜇implant positioning was selected according to the previous study in which a self-drilled puncture was used to get a pinpoint hole and remove the effect of heavy mucosa and facilitate the insertion of the implant. It was inserted in the area near the mesial aspect of the left maxillary incisor to pull the spring a distance to generate approximately 50 g of orthodontic force.

**Figure 1:**
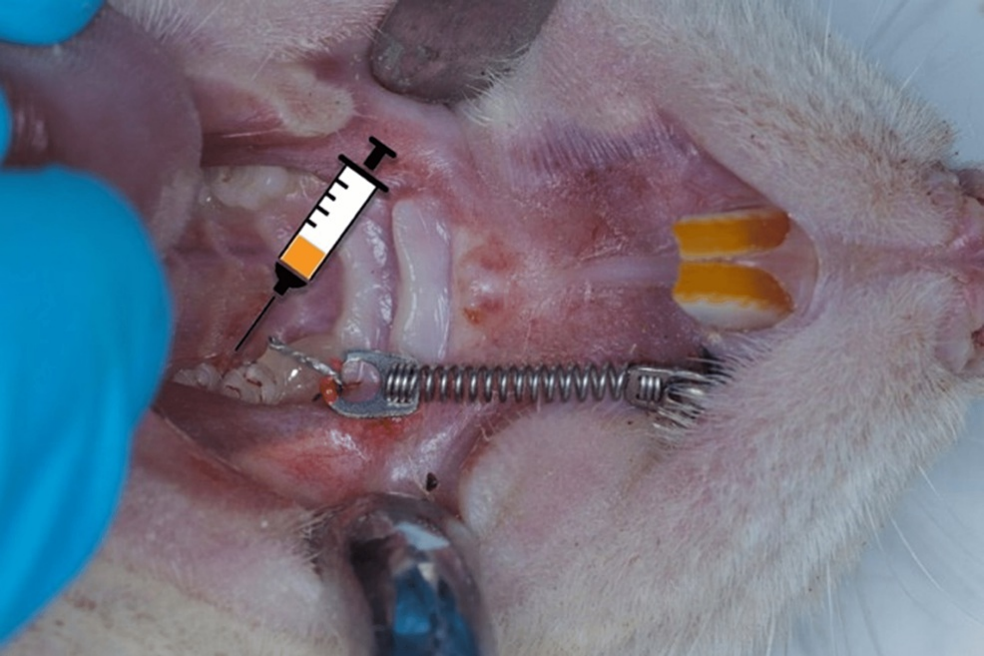
A schematic drawing of the proposed rat model

To prevent trauma and slippage of the wire, the end of the wire and the head of 𝜇implant were covered with flowable composite and light cured. This device permits the upper left first molar to slide mesially away from the distal molars. The appliance remained in place for three weeks in order to induce significant tooth movement (Figure 1). The left side of each animal’s maxilla, which acted as the experimental side, received orthodontic therapy, whereas the right side received no treatment. All the procedures and measurements were carried out under general anesthesia. At each time point, the stability of the orthodontic appliance was checked for any dislodgment.

Animals grouping and treatment timing

Eighty male rats were divided into two groups of 40 rats then each group was subdivided into four subgroups: 1, control; 2, injected with 75 µg/kg of CaCO_3_ NPs; 3, injected with 5 µg/kg of rhBMP-2; and 4, injected with 80 µg/kg of CaCO_3_ NPs-rhBMP-2 subgroups. The total experiment duration lasted for 9 weeks, the first 3-week phase was for active OTM applied to all experimental groups. Then, in the second 3-week phase, the animals were randomly divided into two experimental groups. The first group (6 weeks), in which active orthodontic appliance was removed along with the administration of nano biological substances weekly. The second group (9 weeks), in which active orthodontic appliance was replaced with passive ligation for the second 3 weeks along with the administration of nano biological substances weekly was followed by the third 3-week phase in which passive ligation was removed for relapse.

Administration of nano biological substances

Administration of therapies was performed during the second 3-week phase for all of the subgroups. A pre-prepared substance, designed for each group, was injected distal to the mesially moved tooth at three-time points. The 1st, 2nd, and 3rd doses were injected on days 21, 28, and 35, respectively.

Measurements of animals’ body weight

In order to monitor the effect of the orthodontic appliance with and without nano biological retainers on animals’ BW changes, it was measured o 0, 21, 28, 35, and 42 days for both groups and also at 63 days for group 2 (9 weeks).

Statistical analysis

Statistical analysis was carried out by using the SPSS version. The data were described in min, max, and mean±SD. One-way analysis of variance (ANOVA) for BW difference in mean changes across time and groups was compared. It was determined to be significant if P was less than 0.05. Following that, differences that were found to be statistically significant were analyzed further using post hoc (Tukey’s test) for multiple comparisons.

## Results

The demographic description of the two experimental groups (6-week and 9-week) include means, standard deviations, and minimum and maximum of the BW measured in gram (g); at all testing time points were shown in Table 1.

**Table 1:** The descriptive Statistics of the 6-week and 9-week sets

		6-week groups					9-week groups			
Body Weight (gm)	N	Minimum	Maximum	Mean	Std. Dev	N	Minimum	Maximum	Mean	Std. Dev
BW_0 day (gm)	31	325.00	475.00	403.77	47.28	30	350.00	475.00	408.33	40.70
BW_21 days (gm)	31	302.50	447.50	377.92	45.18	30	326.50	444.00	385.63	38.81
BW_28 days (gm)	31	298.50	449.25	375.57	46.95	30	303.00	432.25	365.36	43.00
BW_35 days (gm)	31	279.00	431.00	355.39	45.68	30	289.75	413.00	348.59	41.51
BW_42 days (gm)	31	251.25	398.50	325.08	44.11	30	283.50	395.25	335.16	38.37
BW_63 days (gm)						30	266.00	373.75	312.27	35.91

The normality test for the collected data was conducted using the Shapiro-Wilk test which showed that the vast majority of the variables were normally distributed; therefore, a parametric test (2-way ANOVA) was used.

**Figure 2:**
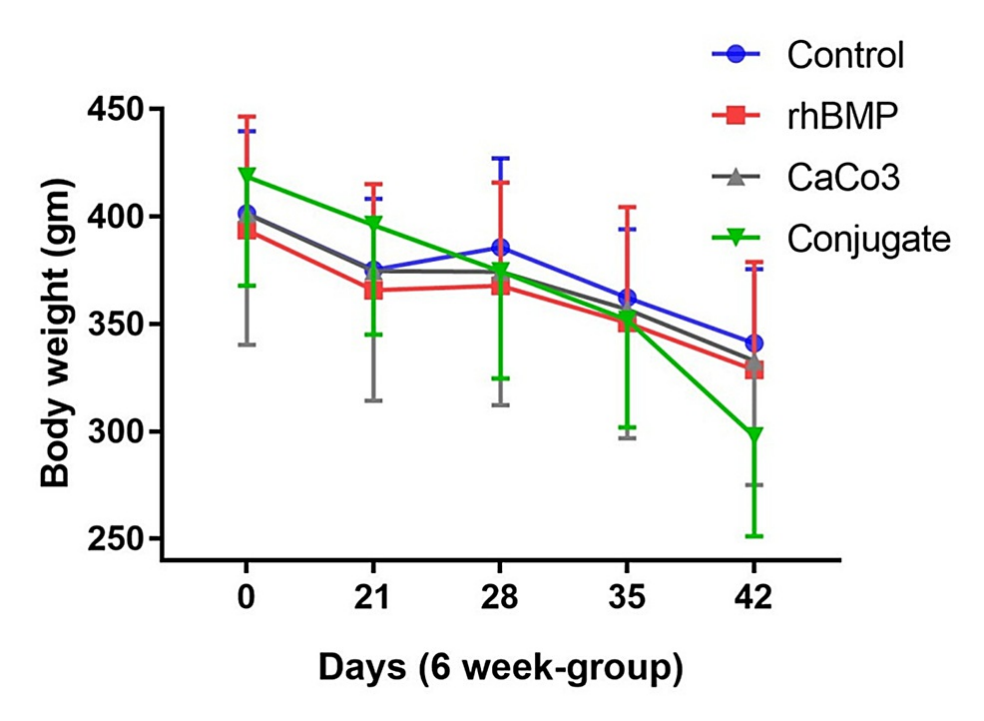
The body weight of the four animal subgroups (of the 6-week set) across the time (42 days) gm: gram; rhBMP: recombinant bone morphogenetic protein; CaCO_3_: calcium carbonate

**Figure 3:**
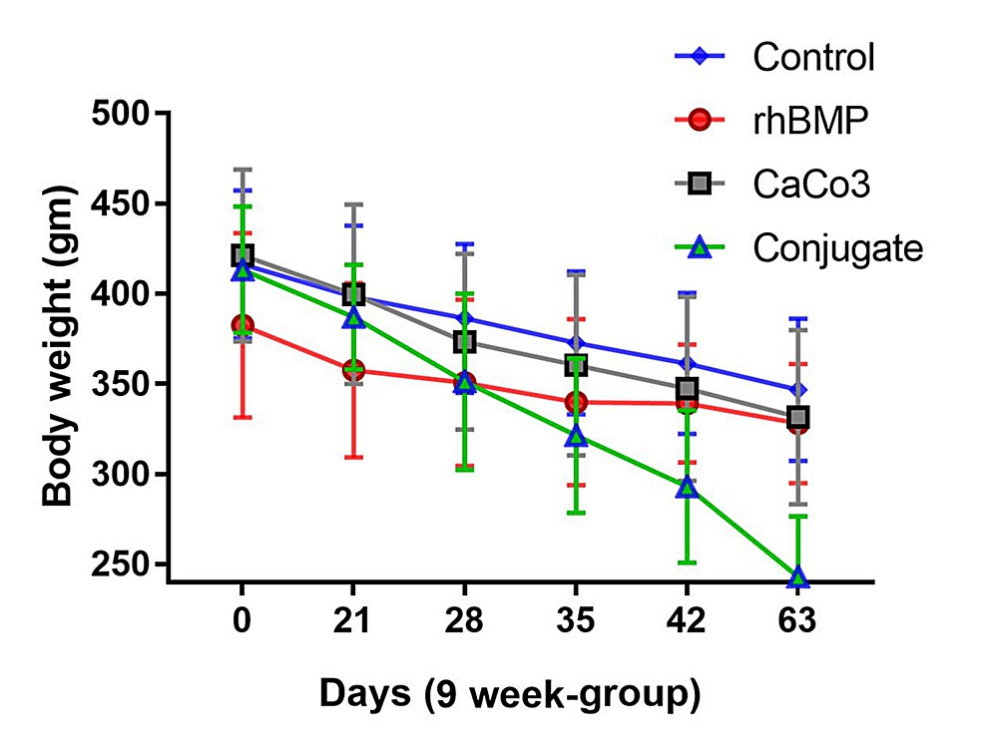
The body weight of the four animal subgroups (of the 9-week set) across the time (63 days) gm: gram; rhBMP: recombinant bone morphogenetic protein; CaCO_3_: calcium carbonate

The BW of animals in both sets (6- and 9-week) reduced significantly over time and the weight of the conjugate (CaCO3+BMP) group had the highest reduction over time (Figures 2-3). The reduction in the average BW of the four groups in the 9-week set was higher than the reduction in the average BW of the four groups in the 6-week set. Additionally, the reduction in the average BW of the conjugate group in the 9-week set was higher than that in the 6-week set on the 42nd day (Figures 2-3).

However, there were no significant (P-value ˃0.05) differences in the BW between the groups of the two (6-week and 9-week) sets (Tables 2-3) and within the subgroups of the 6-week set across each time point (Table 4).

**Table 2:** Tukey’s multiple comparisons for BW between 6-week subgroups at each time point ns: non-significant; sig: significant; Diff: difference; gm: gram; rhBMP: recombinant bone morphogenetic protein; CaCO_3_: calcium carbonate; BW: body weight

6-week subgroups	Mean Diff.	95.0% CI of diff.	Sig.	P-Value
BW-Day 0				
Control vs. BMP	7.75	-57.24 to 72.74	ns	0.989
Control vs. CaCO_3_	0.25	-64.74 to 65.24	ns	0.999
Control vs. conjugate	-17.07	-84.34 to 50.2	ns	0.912
BMP vs. CaCO_3_	-7.50	-72.49 to 57.49	ns	0.991
BMP vs. conjugate	-24.82	-92.09 to 42.45	ns	0.772
CaCO_3_ vs. conjugate	-17.32	-84.59 to 49.95	ns	0.908
BW-Day 21				
Control vs. BMP	9.50	-55.49 to 74.49	ns	0.981
Control vs. CaCO_3_	0.63	-64.36 to 65.61	ns	0.999
Control vs. conjugate	-20.75	-88.02 to 46.52	ns	0.853
BMP vs. CaCO_3_	-8.88	-73.86 to 56.11	ns	0.984
BMP vs. conjugate	-30.25	-97.52 to 37.02	ns	0.647
CaCO_3_ vs. conjugate	-21.38	-88.64 to 45.89	ns	0.842
BW-Day 28				
Control vs. BMP	17.75	-47.24 to 82.74	ns	0.893
Control vs. CaCO_3_	11.25	-53.74 to 76.24	ns	0.969
Control vs. conjugate	11.20	-56.07 to 78.46	ns	0.973
BMP vs. CaCO_3_	-6.50	-71.49 to 58.49	ns	0.994
BMP vs. conjugate	-6.55	-73.82 to 60.71	ns	0.994
CaCO_3_ vs. conjugate	-0.05	-67.32 to 67.21	ns	0.999
BW-Day 35				
Control vs. BMP	11.75	-53.24 to 76.74	ns	0.965
Control vs. CaCO_3_	5.38	-59.61 to 70.36	ns	0.996
Control vs. conjugate	10.39	-56.87 to 77.66	ns	0.978
BMP vs. CaCO_3_	-6.38	-71.36 to 58.61	ns	0.994
BMP vs. conjugate	-1.36	-68.62 to 65.91	ns	0.999
CaCO_3_ vs. conjugate	5.09	-62.25 to 72.29	ns	0.997
BW-Day 42				
Control vs. BMP	12.50	-52.49 to 77.49	ns	0.959
Control vs. CaCO_3_	8.25	-56.74 to 73.24	ns	0.988
Control vs. conjugate	43.41	-23.86 to 110.7	ns	0.339
BMP vs. CaCO_3_	-4.25	-69.24 to 60.74	ns	0.998
BMP vs. conjugate	30.91	-36.36 to 98.18	ns	0.631
CaCO_3_ vs. conjugate	35.16	-32.11 to 102.4	ns	0.527

**Table 3:** Tukey’s multiple comparisons for BW between 9-week subgroups at each time point *significant ns: non-significant; sig: significant; Diff: difference; gm: gram; rhBMP: recombinant bone morphogenetic protein; CaCO_3_: calcium carbonate; BW: body weight

9-week subgroups	Mean Diff.	95.00% CI of diff.	Sig.	P-Value
BW-Day 0				
Control vs. BMP	33.75	-22.69 to 90.19	ns	0.4087
Control vs. CaCO_3_	-5	-61.44 to 51.44	ns	0.9957
Control vs. conjugate	2.917	-58.05 to 63.88	ns	0.9993
BMP vs. CaCO_3_	-38.75	-95.19 to 17.69	ns	0.2854
BMP vs. conjugate	-30.83	-91.8 to 30.13	ns	0.5558
CaCO_3_ vs. conjugate	7.917	-53.05 to 68.88	ns	0.9867
BW-Day 21				
Control vs. BMP	40.63	-15.82 to 97.07	ns	0.2456
Control vs. CaCO_3_	-1.375	-57.82 to 55.07	ns	>0.9999
Control vs. conjugate	11.25	-49.72 to 72.22	ns	0.9636
BMP vs. CaCO_3_	-42	-98.44 to 14.44	ns	0.2188
BMP vs. conjugate	-29.38	-90.34 to 31.59	ns	0.5953
CaCO_3_ vs. conjugate	12.63	-48.34 to 73.59	ns	0.9497
BW-Day 28				
Control vs. BMP	35.63	-20.82 to 92.07	ns	0.3598
Control vs. CaCO_3_	12.88	-43.57 to 69.32	ns	0.9343
Control vs. conjugate	35.08	-25.88 to 96.05	ns	0.4433
BMP vs. CaCO_3_	-22.75	-79.19 to 33.69	ns	0.7222
BMP vs. conjugate	-0.5417	-61.51 to 60.42	ns	>0.9999
CaCO_3_ vs. conjugate	22.21	-38.76 to 83.17	ns	0.7801
BW-Day 35				
Control vs. BMP	32.88	-23.57 to 89.32	ns	0.4324
Control vs. CaCO_3_	12.38	-44.07 to 68.82	ns	0.941
Control vs. conjugate	51.42	-9.549 to 112.4	ns	0.1305
BMP vs. CaCO_3_	-20.5	-76.94 to 35.94	ns	0.7816
BMP. conjugate	18.54	-42.42 to 79.51	ns	0.859
CaCO_3_ vs conjugate	39.04	-21.92 to 100	ns	0.3468
BW-Day 42				
Control vs. BMP	22.13	-34.32 to 78.57	ns	0.7392
Control vs. CaCO_3_	14	-42.44 to 70.44	ns	0.9175
Control vs. conjugate	68.25	7.284 to 129.2	*	0.0215
BMP vs. CaCO_3_	-8.125	-64.57 to 48.32	ns	0.9821
BMP vs. conjugate	46.13	-14.84 to 107.1	ns	0.2059
CaCO_3_ vs. conjugate	54.25	-6.716 to 115.2	ns	0.0999
BW-Day 63				
Control vs. BMP	18.63	-37.82 to 75.07	ns	0.8269
Control vs. CaCO_3_	15	-41.44 to 71.44	ns	0.9007
Control vs. conjugate	103.8	42.83 to 164.8	*	0.0001
BMP vs. CaCO_3_	-3.625	-60.07 to 52.82	ns	0.9983
BMP vs. conjugate	85.17	24.2 to 146.1	*	0.0022
CaCO_3_ vs. conjugate	88.79	27.83 to 149.8	*	0.0013

**Table 4:** Tukey’s multiple comparisons for BW within each subgroup of the 6-week group at different time points *significant ns: non-significant; sig: significant; Diff: difference; gm: gram; rhBMP: recombinant bone morphogenetic protein; CaCO_3_: calcium carbonate; BW: body weight

Time points—6 weeks	Mean Diff.	95.00% CI of diff.	Sig.	P-Value
Control				
0 vs. 21	26.25	5.713 to 46.79	*	0.0048
0 vs. 28	15.88	-4.662 to 36.41	ns	0.2126
0 vs. 35	39.25	18.71 to 59.79	*	<0.0001
0 vs. 42	60.38	39.84 to 80.91	*	<0.0001
21 vs. 28	-10.38	-30.91 to 10.16	ns	0.6348
21 vs. 35	13	-7.537 to 33.54	ns	0.4108
21 vs. 42	34.13	13.59 to 54.66	*	<0.0001
28 vs. 35	23.38	2.838 to 43.91	*	0.0168
28 vs. 42	44.5	23.96 to 65.04	*	<0.0001
35 vs. 42	21.13	0.5879 to 41.66	*	0.0404
BMP				
0 vs. 21	28	7.463 to 48.54	*	0.0021
0 vs. 28	25.88	5.338 to 46.41	*	0.0057
0 vs. 35	43.25	22.71 to 63.79	*	<0.0001
0 vs. 42	65.13	44.59 to 85.66	*	<0.0001
21 vs. 28	-2.125	-22.66 to 18.41	ns	0.9986
21 vs. 35	15.25	-5.287 to 35.79	ns	0.2492
21 vs. 42	37.13	16.59 to 57.66	*	<0.0001
28 vs. 35	17.38	-3.162 to 37.91	ns	0.1402
28 vs. 42	39.25	18.71 to 59.79	*	<0.0001
35 vs. 42	21.88	1.338 to 42.41	*	0.0305
CaCO_3_				
0 vs. 21	26.63	6.088 to 47.16	*	0.004
0 vs. 28	26.88	6.338 to 47.41	*	0.0036
0 vs. 35	44.38	23.84 to 64.91	*	<0.0001
0 vs. 42	68.38	47.84 to 88.91	*	<0.0001
21 vs. 28	0.25	-20.29 to 20.79	ns	>0.9999
21 vs. 35	17.75	-2.787 to 38.29	ns	0.1254
21 vs. 42	41.75	21.21 to 62.29	*	<0.0001
28 vs. 35	17.5	-3.037 to 38.04	ns	0.1351
28 vs. 42	41.5	20.96 to 62.04	*	<0.0001
35 vs. 42	24	3.463 to 44.54	*	0.013
Conjugate				
0 vs. 21	22.57	0.6164 to 44.53	*	0.0406
0 vs. 28	44.14	22.19 to 66.1	*	<0.0001
0 vs. 35	66.71	44.76 to 88.67	*	<0.0001
0 vs. 42	120.9	98.9 to 142.8	*	<0.0001
21 vs. 28	21.57	-0.3836 to 43.53	ns	0.0568
21 vs. 35	44.14	22.19 to 66.1	*	<0.0001
21 vs. 42	98.29	76.33 to 120.2	*	<0.0001
28 vs. 35	22.57	0.6164 to 44.53	*	0.0406
28 vs. 42	76.71	54.76 to 98.67	*	<0.0001
35 vs. 42	54.14	32.19 to 76.1	*	<0.0001

In contrast, there was a significant (P-value ˂0.05) difference between the BW of the conjugate subgroup and the other three subgroups in the 9-week set, particularly on 63rd day (Table 5). On the other hand, there was a significant (P-value ˂0.05) reduction in the BW over time in both sets.

**Table 5:** Tukey’s multiple comparisons for BW within each subgroup of the 9-week group at different time points *significant ns: non-significant; sig: significant; Diff: difference; gm: gram; rhBMP: recombinant bone morphogenetic protein: CaCO_3_: calcium carbonate; BW: body weight

Time points—9 week	Mean Diff.	95.00% CI of diff.	Sig.	P-Value
Control				
0 vs. 21	18	-10.03 to 46.03	ns	0.433
0 vs. 28	30	1.971 to 58.03	*	0.0284
0 vs. 35	43.5	15.47 to 71.53	*	0.0002
0 vs. 42	55	26.97 to 83.03	*	<0.0001
0 vs. 63	69.63	41.6 to 97.65	*	<0.0001
21 vs. 28	12	-16.03 to 40.03	ns	0.8171
21 vs. 35	25.5	-2.529 to 53.53	ns	0.097
21 vs. 42	37	8.971 to 65.03	*	0.0028
21 vs. 63	51.63	23.6 to 79.65	*	<0.0001
28 vs. 35	13.5	-14.53 to 41.53	ns	0.731
28 vs. 42	25	-3.029 to 53.03	ns	0.1096
28 vs. 63	39.63	11.6 to 67.65	*	0.001
35 vs. 42	11.5	-16.53 to 39.53	ns	0.8424
35 vs. 63	26.13	-1.904 to 54.15	ns	0.0829
42 vs. 63	14.63	-13.4 to 42.65	ns	0.6589
BMP				
0 vs. 21	24.88	-3.154 to 52.9	ns	0.113
0 vs. 28	31.88	3.846 to 59.9	*	0.016
0 vs. 35	42.63	14.6 to 70.65	*	0.0003
0 vs. 42	43.38	15.35 to 71.4	*	0.0002
0 vs. 63	54.5	26.47 to 82.53	*	<0.0001
21 vs. 28	7	-21.03 to 35.03	ns	0.9789
21 vs. 35	17.75	-10.28 to 45.78	ns	0.4491
21 vs. 42	18.5	-9.529 to 46.53	ns	0.4012
21 vs. 63	29.63	1.596 to 57.65	*	0.0317
28 vs. 35	10.75	-17.28 to 38.78	ns	0.8767
28 vs. 42	11.5	-16.53 to 39.53	ns	0.8424
28 vs. 63	22.63	-5.404 to 50.65	ns	0.1878
35 vs. 42	0.75	-27.28 to 28.78	ns	>0.9999
35 vs. 63	11.88	-16.15 to 39.9	ns	0.8236
42 vs. 63	11.13	-16.9 to 39.15	ns	0.8601
CaCO_3_				
0 vs. 21	21.63	-6.404 to 49.65	ns	0.2305
0 vs. 28	47.88	19.85 to 75.9	*	<0.0001
0 vs. 35	60.88	32.85 to 88.9	*	<0.0001
0 vs. 42	74	45.97 to 102	*	<0.0001
0 vs. 63	89.63	61.6 to 117.7	*	<0.0001
21 vs. 28	26.25	-1.779 to 54.28	ns	0.0803
21 vs. 35	39.25	11.22 to 67.28	*	0.0012
21 vs. 42	52.38	24.35 to 80.4	*	<0.0001
21 vs. 63	68	39.97 to 96.03	*	<0.0001
28 vs. 35	13	-15.03 to 41.03	ns	0.7612
28 vs. 42	26.13	-1.904 to 54.15	ns	0.0829
28 vs. 63	41.75	13.72 to 69.78	*	0.0005
35 vs. 42	13.13	-14.9 to 41.15	ns	0.7538
35 vs. 63	28.75	0.7207 to 56.78	*	0.0409
42 vs. 63	15.63	-12.4 to 43.65	ns	0.5918
Conjugate				
0 vs. 21	26.33	-6.032 to 58.7	ns	0.1807
0 vs. 28	62.17	29.8 to 94.53	*	<0.0001
0 vs. 35	92	59.63 to 124.4	*	<0.0001
0 vs. 42	120.3	87.97 to 152.7	*	<0.0001
0 vs. 63	170.5	138.1 to 202.9	*	<0.0001
21 vs. 28	35.83	3.468 to 68.2	*	0.0208
21 vs. 35	65.67	33.3 to 98.03	*	<0.0001
21 vs. 42	94	61.63 to 126.4	*	<0.0001
21 vs. 63	144.2	111.8 to 176.5	*	<0.0001
28 vs. 35	29.83	-2.532 to 62.2	ns	0.0892
28 vs. 42	58.17	25.8 to 90.53	*	<0.0001
28 vs. 63	108.3	75.97 to 140.7	*	<0.0001
35 vs. 42	28.33	-4.032 to 60.7	ns	0.1224
35 vs. 63	78.5	46.13 to 110.9	*	<0.0001
42 vs. 63	50.17	17.8 to 82.53	*	0.0002

There was no significant difference among the four groups of the 6-week set according to the animal BW (Table 2) and the 9-week set (Table 3).

Within each group, the animal BW reduced significantly after the intervention. In other words, the animal weight was significantly lower after treatment compared to before the intervention (time zero) within the four groups of the 6-week set (Table 4) and the 9-week set (Table 5).

**Figure 4:**
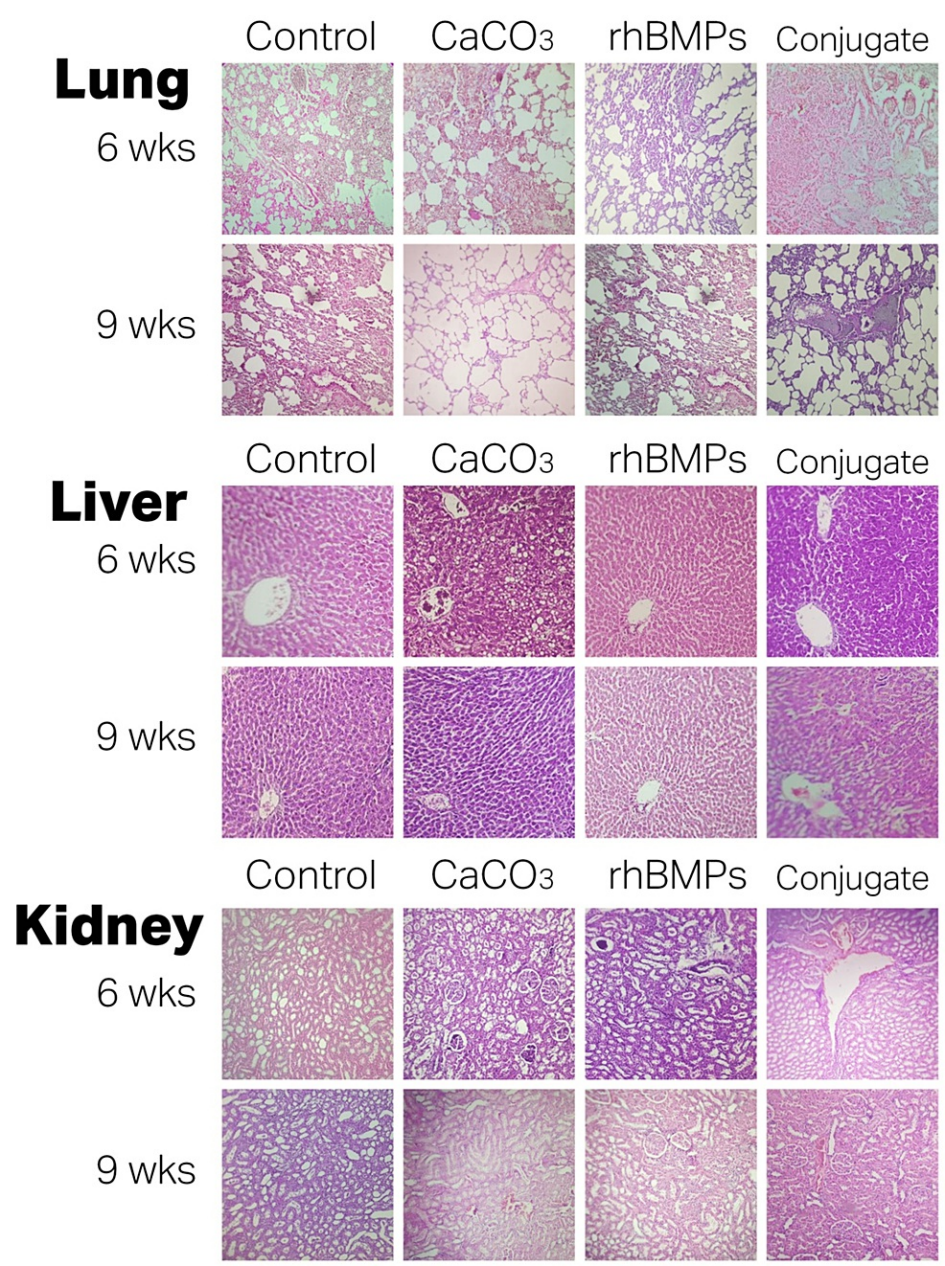
Histological findings of organs Micrographs of the lung, liver, and kidney after 6 and 9 weeks of treatment, H&E (X10). wks: weeks; rhBMP: recombinant bone morphogenetic protein; CaCO_3_: calcium carbonate

Histologically, in the conjugated group, severe inflammation changes were seen in the lungs, including different stages of inflammation hemorrhage (Figure 4). However, in BMP group, mild to moderate cloudy swelling was observed in the kidney. In the CaCO_3_ group, mild congestions were found in the liver.

## Discussion

Rats were used as animal model for several biological and practical advantages, for example, the animal is relatively inexpensive, tissue changes incident to OTM are similar but faster in rats than humans, also most antibodies required for cellular and molecular biological techniques are relatively inexpensive and available for rat and mice [15,16].

Without a doubt, many issues should be considered with the presence of fixed orthodontic appliances in the mouth of humans as well as in animals. It highly interferes with normal eating and swallowing resulting in a limited amount of food intake. Additionally, chewing and swallowing hard food becomes difficult and the masticatory ability is reduced after inserting a fixed orthodontic appliance [17]. Moreover, in order to preserve the fixed orthodontic appliance during the whole treatment period, the food texture should be changed to soft food and avoid any hard diet that may cause a fracture of the inserted fixed appliance. The results of this study showed that the BW of animals in all studied groups was reduced significantly over time which comes in agreement with the results of previous studies that also found a reduction of BW of experimental rats following the movement of teeth using fixed orthodontic appliance although some variations were observed which could be related to the other cofounders of each study [12,18]. This reduction in BW could be related to the abovementioned issues which associated with the insertion of an orthodontic appliance that may induce pain and interfere with normal eating resulting in a decrease in BW that is directly proportional to the presence of an orthodontic appliance which was confirmed by more weight loss in 9-week group compared to the 6-week group.

Furthermore, the animal weight was significantly lower after injecting the biological substance compared to that before injection (time zero) which means that BW was reduced significantly after biological intervention with the highest reduction observed in the conjugate (CaCO_3_+BMP) group. This may be related to the effect of the injected substances (CaCO_3_ and/or BMP) resulting in the interruption in absorption and metabolism of feed nutrients essential for the health of rats. For example, it was reported that the BW of rats treated with calcium as 50 mg/kg BW was gradually decreased but not statistically significant up to 42 consecutive days of treatment [19]. Other studies, their results indicated a non-significant decrease in the percentage of BW gain in premature rats fed on high-calcium diets while a significant decrease in the percentage of BW gain in mature rats fed on the same diet composition [20,21]. Additionally, BMPs are important regulators of energy balance, including those processes regulated by the nervous system, such as hypothalamic appetite control and sympathetic outflow to adipose depots to regulate lipolysis, browning and thermogenesis. Taken together with the knowledge that neural plasticity, including in the energy balance center of the brain-the hypothalamus-is important for metabolism and BW regulation, it is very likely that BMPs in the adult brain are mediating changes to neural plasticity that have impacts on energy balance. This likely underlies observations in the literature where compensation of BMP activities in the brain led to restored energy balance. Given that BMP ligands such as BMP7 can decrease appetite as well as promote energy expenditure and browning (all of which improve metabolic outcomes) [22].

Limitation of the study

In spite of a highly standardized procedure including animal selection, housing, orthodontic technique, and materials used to move teeth, the individual variation between animals included in this study cannot be omitted such as general body health and their response to the applied orthodontic force, also the pain threshold of each animal and its effect on feeding. Similarly, the body organ responses to the injected substances differ individually. Therefore, further studies are recommended with a larger sample size to minimize the individual variation and more specific histological examination of body organs to specify the precise effect of the injected biological substances.

## Conclusions

The current study concluded that animal BW decrease following the insertion of a fixed orthodontic appliance in rat’s mouth as it affects normal eating and this weight loss enhanced with the local injection of a biological substance that was used to improve OTM such as rhBMP, CaCO_3_ individually or synergistically. However, histological examination for general body organs (lung, liver, and kidney) showed mild acceptable changes when used either rhBMP or CaCO_3_ alone however severe changes when used in the conjugate.
